# A patient with a homozygous diacylglycerol kinase epsilon (DGKE) gene mutation with atypical haemolytic uraemic syndrome and low C3 responded well to eculizumab: a case report

**DOI:** 10.1186/s12882-021-02352-8

**Published:** 2021-04-20

**Authors:** Muneera Alabdulqader, Khalid Alfakeeh

**Affiliations:** 1 Department of Paediatrics, College of Medicine, King Faisal University, Alhasa, Saudi Arabia; 2 Department Paediatric Nephrology, King Abdullah Specialists Children Hospital, Riyadh, Saudi Arabia

**Keywords:** Haemolytic uraemic syndrome, DGKE mutation, Eculizumab

## Abstract

**Background:**

Atypical haemolytic uraemic syndrome (aHUS) is a rare systemic syndrome characterized by non-immune haemolytic anaemia, thrombocytopenia, and kidney injury. In most cases, alternative complement pathway dysregulation is the identifying cause. Recently, other genetic causes have been identified, including a mutation in the diacylglycerol kinase epsilon (DGKE) gene, which theoretically affect the coagulation pathway and does not affect the complement pathway. Data about the management of these patients are limited. Ideal management and definitive treatment protocols have not yet been established.

**Case presentation:**

A three-year-old boy presented with features of atypical haemolytic uraemic syndrome (aHUS) and low complement C3. He was presumed to have complement-mediated aHUS and was managed empirically with eculizumab. Two weeks after starting eculizumab, his haemoglobin levels, platelet count, and complement C3 level normalized but he continued to have non-nephrotic range proteinuria. His genetic testing revealed a homozygous DGKE mutation, with no other mutation detected. Six months after presentation, the patient was still in remission with no features of aHUS, a trial of weaning eculizumab by increasing dose interval was followed by nephrotic range proteinuria and severe oedema. His proteinuria improved and his oedema resolved after resuming his recommended eculizumab dose.

**Conclusions:**

DGKE gene mutation can lead to aHUS with theoretically no complement dysregulation. However, some patients with this mutation show alternative complement pathway activation. This case report describes a patient with aHUS due to a DGKE gene mutation and low C3 levels who responded to eculizumab, adding to the previously reported cases of patients with DGKE gene mutations who had complete remission with no relapse with C5 blockers and/or plasma exchange. A randomized controlled study on patients with DGKE mutations might be beneficial in understanding the disease and generating a management protocol.

## Background

Atypical haemolytic uraemic syndrome (aHUS) is a rare systemic syndrome characterized by non-immune haemolytic anaemia, thrombocytopenia, and kidney injury. In most cases, alternative complement pathway dysregulation is the identifying cause [1]. Approximately 70 % of patients diagnosed with aHUS have a mutation in genes coding alternative complement pathway regulatory factors, including loss-of-function mutations in (complement factor H, complement factor I, or CD46) or gain-of-function mutations in (complement factor B or complement factor 3). Another 10–12 % have antibodies against complement factor H, which eventually leads to the overactivation of the alternative pathway [1]. Other genetic mutations have been identified with the increased use of whole-exome sequencing to investigate patients who had no known mutation. A small number of patients with aHUS have a mutation in genes affecting the coagulation pathway, which theoretically does not affect the complement pathway. Lemaire et al. [2] first described early-onset aHUS cases associated with homozygous or compound heterozygous mutations in the diacylglycerol kinase epsilon (DGKE) gene. Mutations in DGKE cause the overactivation of protein kinase C (PKC), which increases thrombus formation [2].

## Case presentation

A now four-year-old boy presented at the age of 3 years to our emergency department with a 2-week history of fever (39.5 °C), vomiting, and watery stools, which had already improved. He also had lower limb oedema and periorbital puffiness for one week. Apart from receiving paracetamol for the fever, there was no history of medication use. He is the offspring of parents in a consanguineous marriage with an unremarkable past medical history. He has one sister and three brothers. One of his brothers was diagnosed with steroid-resistant nephrotic syndrome due to membranous proliferative glomerulonephritis (MPGN) with C3 deposition diagnosed by kidney biopsy at 3 years of age. He reached chronic kidney disease stage 5 at the age of 4 years. Dialysis was considered, but he died in another hospital due to sepsis. No genetic testing was done on his brother.

On initial examination, the patient was stable but hypertensive with blood pressure reading of 136/83 mmHg, generalized oedema and a distended abdomen. No other abnormalities detected in systemic examinations. Laboratory investigations were performed and revealed the following: serum creatinine (45 µmol/L; normal 2.6–52.2 µmol/L), blood urea nitrogen (14.1 mmol/L; normal 1.8–6.4 mmol/L), potassium (5.5 mmol/L), sodium (135 mmol/L), chloride (110 mmol/L), bicarbonate (18 mmol/L), albumin (1.2 g/dL; normal 3.4-4.2 g/dL), haemoglobin (Hgb) (5.2 g/dL; normal 11.5–14.5 g/dL), white blood cell (WBC) count (9.13 × 10^9^/L; normal 4–12 × 10^9^/L), platelet count (23 × 10^9^/L; normal 150–400 × 10^9^/L), haptoglobin (< 0.058 g/L; normal 0.5–2.2 g/L), lactate dehydrogenase (LDH) (1425 U/L; normal 150–500 U/L), complement C3 (C3) (0.550 g/L; normal 0.80–1.60 g/L), and complement C4 (C4) (0.109/L; normal 0.16–0.48 g/L). The patient also had normal coagulation profile, normal level of ADAMTS13, negative coombs test and peripheral blood smears showed schistocytes. His urine dipstick showed urine protein excretion of > 400 mg/dL and 20–50 red blood cells/high power field. Stool cultures and analysis revealed no pathogens (Table 1). Due to his uncontrolled hypertension and thrombocytopenia, the patient could not undergo a kidney biopsy.

**Table 1:** Patient’s laboratory results at different time points

Time	Haemoglobin level (reference range 11.5–14.5 g/dL)	Platelet count (reference range 150–400 × 10^9^/L)	C3 level (reference range 0.80–1.60 g/L)	Protein excretion in first-morning urine dipstick (reference range < 30 mg/dL)
At time of diagnosis and eculizumab initiation	5.9	23	0.55	> 400
1 month after diagnosis	9	169	0.91	100
6 months on eculizumab every 2 weeks	11.8	543	NA	100
1 month after increasing the eculizumab interval to every 3 weeks	12.2	494	0.78	300
4 months after increasing the eculizumab interval to every 3 weeks	11.1	462	NA	300
1 month after resuming eculizumab every 2 weeks	12.2	491	NA	100
4 months after resuming eculizumab every 2 weeks	11	641	NA	100

The patient’s clinical picture fit the diagnosis of aHUS, and eculizumab was started within 24 h of the diagnosis. Following the recommendations of the Food and Drug Administration (FDA) and the manufacturer, he was given a 600 mg intravenous (IV) infusion as induction therapy and a 300 mg IV infusion every two weeks as maintenance therapy. After two doses of eculizumab, he started to show clinical and laboratory improvement. His Hgb improved to 9 g/dL; his platelet count increased to 169 × 10^9^/L; and his C3, haptoglobin, and LDH levels were normalized (Table 1). His blood pressure was controlled on amlodipine and lisinopril, and his oedema significantly improved. A genetic panel for aHUS was performed, and the patient showed a homozygous nonsense gene mutation in DGKE p.(Phe250Serfs*3). No mutation was detected in the gene coding regions of *ADAMTS13, C3, CD46, CFB, CFH, CFHR1, CFHR2, CFHR3, CFHR5, CFI, MMACHC, PIGA, PLG, THBD, CD59, CR1, CR2, INF2*, or *MUT.*

Six months after presentation, the patient was stable and in clinical remission on eculizumab 300 mg every other week. His laboratory parameters were within the normal ranges (Hgb 11.8 g/dL, platelet count 543 × 10^9^/L, C3 0.9 g/L) apart from persistent moderate proteinuria, with urine dipstick 100 mg/dL and serum albumin 2.5 g/dL (Table 1). Based on the genetic results and the patient’s general condition, we decided to start increasing the time between eculizumab doses, targeting discontinuation. A 300 mg IV infusion of eculizumab was administered every three weeks instead of every two weeks. Following the first dose after increasing the time between doses, the patient was noticed to be oedematous; his proteinuria in urine dipstick increased to 400 − 300 mg/dL with urine protein/creatinine ratio > 3 mg/mg, his albumin dropped to 1.9 g/dL, and other laboratory parameters, including Hgb and platelet count, were within the normal ranges (Hgb 12.2 g/dl and platelet count 494 × 10^9^/L) (Table 1). His oedema improved after the addition of oral furosemide at 1 mg/kg/dose twice daily. Nevertheless, after 4 months of eculizumab every 3 weeks, the patient’s proteinuria and albumin levels did not improve. The decision was made to try eculizumab every two weeks and observe his proteinuria. Interestingly, his proteinuria improved to 100 mg/dL after only two doses of the two-week regimen, his oedema subsided and serum albumin improved gradually from 1.9 g/L to 2.2 g/L (Table 1). Four months later, patient is stable on eculizumab 300 mg IV infusion every two weeks with no oedema and stable proteinuria and Albumin (Table 1). Throughout his first year after presentation, apart from proteinuria, his creatinine was within the normal range, and he had no signs of HUS activity after the first remission.

## Discussion and conclusion

DGKE nephropathy is an autosomal-recessive disorder caused by a loss-of-function mutation in the DGKE gene (OMIM phenotype number 615,008). The pathogenesis of its deficiency remains to be fully understood. In healthy individuals, DGKE phosphorylates and inactivates arachidonic acid-containing diacylglycerol (AA-DAG), which is a central signalling molecule of the diacylglycerol (DAG) family and an activator of PKC. PKC activation in endothelial cells increases the production of many prothrombotic factors. A loss of DGKE function may result in sustained AA-DAG signalling, causing a prothrombotic state [2]. Additionally, DAGs are important in slit diaphragm function in podocytes; an alteration of their function due to DGKE mutation can cause persistent proteinuria [2]. Most patients with DGKE mutation present in the first year of life, and almost all patients present before the age of 5 years. A total of 80–94 % present with aHUS, while 6–20 % present with early-onset nephrotic syndrome and membrane proliferative glomerulonephritis (MPGN)-like features on kidney biopsy [3, 4]. These patients who present with aHUS have a 64–70 % rate of relapse after initial remission and usually have persistent proteinuria and microscopic haematuria in between relapses [2, 5]. For the last decade, the empirical therapy for patients diagnosed with aHUS has been eculizumab, a recombinant humanized monoclonal antibody that binds to complement component 5 (C5) to inhibit terminal complement cascade activation. If eculizumab therapy is not available, patients are managed by plasma infusions to replace deficient factors or plasmapheresis to remove anti-complement factor H antibodies. While the effectiveness of C5 blockers is well established in complement-medicated HUS, it is still not clear in aHUS due to DGKE mutation [2, 6]. Although DGKE mutation-associated aHUS is not considered to be complement mediated, 19–29 % of patients show evidence of alternative pathway activation, with low C3 levels or increased C5b-9 levels during diagnosis and/or relapses [4]. This raises the question of whether all cases of DGKE mutation-associated aHUS are truly complement independent. Initial reports in aHUS due to DGKE mutation suggested a benefit of plasma infusion and eculizumab therapy. Chinchilla et al.[6] reported the case of a patient with a DGKE mutation who presented with aHUS and low C3 whose condition improved with plasma infusion and eculizumab. Interestingly, this patient had another mutation in C3 combined with a membrane cofactor protein (MPC) risk haplotype in homozygosity. These concomitant mutations can also cause complement-mediated aHUS, which might explain the improvement with eculizumab in this specific case. More reports of patients with DGKE mutations showed transient or persistent improvement since eculizumab was approved [6, 7]. However, the large case review published by Azukaitis et al.[4] included 44 patients with DGKE mutations, and 16 patients had aHUS and received some form of aHUS-specific therapy. Out of these patients, 10 received plasma infusions, and the author attributed the improvement in their clinical picture to the plasma infusions since 4 patients relapsed after the prolongation of the plasma infusion interval and then responded to therapy intensification. The other 6 patients received eculizumab either at initial presentation or during the course of their illness. Three patients showed improvement after starting eculizumab, but 2 relapsed while receiving regular doses of eculizumab, and the other 3 patients showed no improvement. In the follow-up of these patients, it was noticed that the disease course and long-term outcome were similar with and without aHUS-specific therapy [4]. Brocklebank et al. [5] recently reported the long-term outcomes of 16 patients with DGKE mutations. Six out of the 16 patients received plasma infusion/plasma exchange, and one patient received eculizumab during initial presentation. Another 5 were given eculizumab during the course of their illness. Eculizumab was successfully withdrawn from 4 patients, and relapse occurred in one patient 6 weeks after withdrawal and was managed successfully with supportive therapy[5]. In our case, the patient presented with aHUS and low C3. He was managed empirically with eculizumab and showed improvement. After DGKE-mediated aHUS diagnosis and a trial to wean off eculizumab, the patient’s proteinuria worsened, and he developed severe oedema that improved dramatically after resuming his recommended eculizumab dose. It is difficult to establish whether these improvements are related to the treatment modality or the illness’s natural course for two reasons. First, similar to our patient, many patients present with aHUS are managed empirically with eculizumab before genetic results. Second, reports show patients with complement-mediated aHUS successfully weaned off eculizumab, especially patients with no pathological mutations in complement coding regions [8].

Although eculizumab is an effective and safe treatment, it is not without its risks. The most severe adverse event is the increased susceptibility to encapsulated bacterial infections, especially Neisseria meningitidis. The incidence of meningococcal disease can increase up to 2,000-fold in patients receiving eculizumab [9, 10]. Administrating meningococcal vaccine before starting eculizumab can decrease the risk but does not eliminate it. Therefore prophylactic antibiotics can be considered during eculizumab treatment [10, 11]. The other major limitation in using eculizumab is the up to € 500,000 per year per patient cost, which affects its availability in many countries.

As an important implication for the management of DGKE nephropathy, patients who reach end-stage kidney disease (ESKD) due to DGKE mutations show less chance of recurrence after renal transplantation than individuals with complement-mediated aHUS. Fortunately, to date, there was no recurrence in the 6 reported patients with DGKE mutations who reached ESKD and underwent kidney transplantation [4, 12].

In conclusion, DGKE nephropathy is very rare disease. Supportive therapy is the cornerstone of DGKE nephropathy management. Patients with DGKE mutations and low C3 should have a coexisting mutation that might cause complement-medicated aHUS to be ruled out. Furthermore, there are no clear recommendations on whether to use complement-targeted therapy, including plasma infusions or C5 blockers, e.g., eculizumab, in managing such patients. A randomized controlled study on patients with DGKE mutations comparing eculizumab versus supportive therapy alone might be beneficial in understanding the disease and generating a management protocol.

## Data Availability

Datasets used in this article are available from the corresponding author on reasonable request.

## Abbreviations

DGKE: Diacylglycerol kinase epsilon
aHUS: Atypical haemolytic uraemic syndrome
TMA: Thrombotic microangiopathy
PKC: Protein kinase C
MPGN: Membranous proliferative glomerulonephritis
Hgb: Haemoglobin
WBC: White blood cell
LDH: Lactate dehydrogenase
C3: Complement component 3
C4: Complement component 4
FDA: Food and Drug Administration
AA-DAG: Arachidonic acid-containing diacylglycerol
DAG: Diacylglycerol
C5: Complement component 5
MPC: Membrane protein cofactor
ESKD: End-stage kidney disease
